# Effort-reward imbalance and health outcomes in emergency nurses: the mediating role of work–family conflict and intrinsic effort

**DOI:** 10.3389/fpubh.2024.1515593

**Published:** 2025-01-03

**Authors:** Le Tong, Ling Zhu, Hao Zhang, Luying Zhong, Dongmei Diao, Xiaoli Chen, Jianna Zhang

**Affiliations:** ^1^Department of Emergency Medicine and West China School of Nursing, West China Hospital, Sichuan University, Chengdu, China; ^2^Disaster Medical Center, Sichuan University, Chengdu, China

**Keywords:** work-life balance, emergency nursing, mental health, effort-reward imbalance, intrinsic effort

## Abstract

**Background:**

Emergency nurses experience high stress, but the mechanisms linking effort-reward imbalance to health outcomes are unclear. Work–family conflict might mediate this relationship, and intrinsic effort could moderate it. This study aimed to explore these interactions and their impact on nurse health.

**Methods:**

A prospective cross-sectional survey was conducted from 30 tertiary hospitals across 19 provinces in mainland China. Participants completed validated scales to measure effort-reward imbalance, work–family conflict, somatic symptoms, and mental health symptoms. Statistical analyses, including mediation and moderation, were performed using the PROCESS macro to evaluate the relationships between the variables.

**Results:**

In this study, 1,540 emergency nurses were finally included. The analysis revealed that 80.5% of participants reported experiencing effort-reward imbalance, which was significantly associated with increased somatic symptoms and mental symptoms. Work–family conflict was identified as a significant mediator in the relationship between effort-reward imbalance and health outcomes, with a direct effect of effort-reward imbalance on overall symptoms (*β* = 0.554, *p* < 0.001). Additionally, intrinsic effort moderated the impact of effort-reward imbalance on work–family conflict; moderate levels of emotional investment provided protection against burnout, while excessive intrinsic effort heightened the risk of mental health deterioration (*β* = −0.104, *p* < 0.001).

**Conclusion:**

These findings underscored the urgent need for targeted interventions that address both effort-reward imbalance and work–family conflict in healthcare settings. Enhancing workplace conditions and supporting emotional engagement among emergency nurses are essential strategies for improving their well-being and reducing burnout risk. Future research should focus on longitudinal studies to assess the effectiveness of these interventions over time.

## 1 Introduction

Emergency departments (EDs) are essential healthcare units that provide urgent care for patients with acute and life-threatening conditions. The increasing demand for emergency services worldwide has created increasingly complex and high-pressure work environments. Emergency nurses face specific challenges, including high patient turnover, prolonged and irregular working hours, and the necessity to make rapid, informed decisions under significant stress. These factors contribute to the perception of emergency nursing as a high-risk profession, underscoring the need to understand the determinants of nurse well-being (1–3).

Effort-reward imbalance (ERI), as proposed by Siegrist, suggested that a discrepancy between the effort expended at work and the rewards received could lead to adverse health outcomes (4–7). Numerous studies have demonstrated strong associations between ERI and various health issues, including cardiovascular diseases, gastrointestinal disorders, musculoskeletal pain, and mental health challenges (8–11). The demanding nature of emergency nursing rendered this workforce particularly susceptible to experiencing ERI. For instance, a multicenter study in China reported a prevalence of ERI at 59.66% among emergency nurses, with similar rates exceeding 90% in the United States (6, 12, 13). These findings highlighted an urgent need for interventions aimed at mitigating the effects of ERI.

Work–family conflict (WFC) is another significant factor adversely affecting nurse health outcomes. WFC arises when work demands interfere with family responsibilities and is especially pronounced in emergency nursing (14–16). Existing research indicated that both ERI and WFC are independently linked to negative health outcomes. However, the interaction between these factors and their cumulative effected on nurse health remain insufficiently explored. Understanding how ERI and WFC interact is essential for developing comprehensive strategies to enhance nurse well-being and improve patient care quality.

Additionally, intrinsic effort, defined as the emotional and psychological investment that individuals dedicate to their work, may moderate the relationship between ERI and WFC. Emergency nurses often face substantial emotional demands, which increase the likelihood of role conflict and WFC. Some studies suggested that intrinsic effort may buffer the effects of ERI on WFC, thereby enhancing job satisfaction and mental health (17, 18). However, excessive emotional investment may lead to overcommitment and increased burnout risk. The existing studies indicated that maintaining an optimal level of intrinsic effort could mitigate the negative impacts of both ERI and WFC, although evidence remains limited, particularly in high-pressure environments such as EDs (19).

Previous research has suggested that WFC may act as a mediator between ERI and health outcomes, as it represents a mechanism through which high work demands negatively impact health by interfering with family responsibilities (16). Similarly, intrinsic effort, which refers to the emotional and psychological investment in work, has been identified as a potential moderator in the relationship between ERI and WFC. Research indicated that intrinsic effort may buffer the negative impact of ERI on WFC by fostering resilience and job satisfaction (20, 21). However, excessive intrinsic effort may lead to overcommitment and burnout (22). This study explored these roles and their potential implications for emergency nurses’ health outcomes.

While the above numerous studies have individually explored the relationship between ERI and health outcomes, as well as WFC and nurse well-being, there is a lack of research that simultaneously examines these factors and their cumulative effects. This study aimed to fill this gap by exploring the interaction between ERI and WFC among emergency nurses, with a particular focused on the mediating role of WFC and the moderating effect of intrinsic effort. We hypothesized that ERI was associated with adverse health outcomes in emergency nurses, with WFC acting as a mediator in this relationship. Furthermore, we proposed that intrinsic effort moderated the relationship between ERI and WFC, potentially buffering its negative effects. By elucidating these relationships, this research sought to provide insights that could inform targeted interventions, ultimately enhancing the well-being of emergency nurses and improving patient care outcomes.

## 2 Methods

### 2.1 Study design and participants

In this manuscript, we adhered to the STROBE checklist (23, 24) to ensure the comprehensive and accurate reporting of our cross-sectional observational study. The research was designed as a cross-sectional survey and was a prospective multicenter study.

This study was conducted between Dec. 1st 2023 and Jan. 30th 2024 in EDs of 30 tertiary hospitals across 19 provinces in mainland China.

Stratified cluster sampling was used to select public hospitals across China. Based on a test level of 0.05 and a test efficiency of 80%, the required sample size for the preliminary pre survey calculation was 1,330 cases.

#### 2.1.1 Inclusion and exclusion criteria

The inclusion criteria for our study were as follows: (a) Registered nurses practicing in the ED; (b) Work experience in the ED was ≥1 year; (c) Emergency nursing staff who provided informed consent and voluntarily participate in this study. Exclude ED nurses who had a history of mental illness or were on maternity leave or breastfeeding during the investigation period.

#### 2.1.2 Survey sharing

We applied the Wenjuanxing platform (WJX, China) to create electronic files, which included informed consent forms, questionnaire scales, and generated electronic links. We contacted all the head nurses of EDs in the sampled hospitals. After obtaining consent, the head nurse will distribute a questionnaire link to ED nurses who meet the inclusion criteria through the WeChat network platform (Tencent, China) to conduct a questionnaire survey.

### 2.2 Instruments

#### 2.2.1 Demographic and work characteristics

Demographic data included gender, marital status, number of children, education level, job title, years of work, night shift frequency, weekly working hours, and lifestyle factors (smoking and alcohol using).

#### 2.2.2 Effort-reward imbalance

ERI was assessed using the validated Chinese version of Siegrist’s ERI scale (25, 26). The 22-item scale includes effort (6 items), reward (11 items), and overcommitment (5 items). Each item was rated on a 5-point Likert scale, with higher scores indicating higher effort, lower rewards, or greater overcommitment. The ERI ratio was calculated by dividing the effort score by the reward score, adjusted for item number (effort ÷ [reward × 6/11]). A ratio greater than 1 indicated an ERI.

#### 2.2.3 Symptoms

Symptoms were measured using the Chinese version of the Somatization Symptom Self-Assessment Scale (SSD-CN) (27), a 20-item scale covering four dimensions (somatic complaints, anxiety, depression, and mixed anxiety-depression). Responses were recorded on a 4-point Likert scale. The results could be expressed as overall symptoms, somatic symptoms, and mental symptoms.

The severity of SSD was determined based on the total score: normal was 20–29, mild was 30–39, moderate was 40–50, and severe was ≥60. The test–retest reliability was 0.9. The correlation coefficients between each dimension and the total table were 0.76–0.88, and the correlation coefficients within each dimension were 0.56–0.70. The scale had a cut-off value of 36 points for diagnostic value, a sensitivity of 0.97, and a specificity of 0.96.

#### 2.2.4 Work–family conflict

WFC was measured using the Chinese version of the Work-Family Behavioral Role Conflict Scale (WFBRC-S) (25, 28), which includes 19 items divided into two dimensions: work-to-family conflict and family-to-work conflict. Each item was rated on a 5-point frequency scale, with higher scores indicating more frequent conflict. The maximum score for the scale was 95 points.

### 2.3 Data collection

Data were collected anonymously via an online platform (WeChat) from Dec. 1st 2023 to Jan. 30th 2024. The questionnaires were administered electronically via the WeChat platform (Tencent, China), and all responses were anonymized. Core coordinators at each hospital were trained to ensure consistency in survey administration. Each participant’s response was restricted to one per IP address to avoid duplication. Incomplete or inconsistent responses were excluded from analysis.

### 2.4 Statistical analysis

Statistical analyses were conducted using SPSS 25.0 (SPSS Inc., Chicago, IL, United States). Descriptive statistics were reported as mean (M) ± standard deviation (SD) for continuous variables and as frequencies and percentages for categorical variables. Correlations between ERI, WFC, somatic symptoms, mental health symptoms, and intrinsic effort were examined.

Mediation and moderation analyses were performed using Hayes’ PROCESS macro (29). Mediation was tested using Model 4 to examine whether WFC mediated the relationship between ERI and health outcomes (overall symptoms: somatic and mental symptoms). Moderation was tested using Model 7 to assess whether intrinsic effort moderated the relationship between ERI and WFC. Bootstrap resampling (5,000 iterations) was used to estimate 95% confidence intervals (CI), with statistical significance set at *p* < 0.05.

## 3 Results

### 3.1 Descriptive characteristics

A total of 1,540 valid responses were collected from emergency nurses working in 30 tertiary hospitals across 19 provinces in mainland China. The response rate was 99.04%, as 1,540 valid responses were collected from 1,555 distributed questionnaires. The average age of participants was 32.23 ± 6.80 years (range: 20–58 years), and 78.6% were female. Table 1 provided a detailed summary of the demographic and work-related characteristics.

**Table 1:** Baseline characteristic information of the study population (*n* = 1,540).

Variable	Category	N	%
Gender	Female	1,211	78.6
	Male	329	21.4
Marital status	Unmarried	560	36.4
	Married	980	63.6
Number of children	0	710	46.1
	≥ 1	830	53.9
Educational level	Junior college degree	186	12.1
	Bachelor’s degree or above	1,354	87.9
Job title	Junior professional title	907	58.9
	Intermediate professional title	574	37.3
	Senior professional title	59	3.8
Years of work	1–2	242	15.7
	3–10	742	48.2
	11–20	421	27.3
	Over 20	135	8.8
Night shift	No	181	11.8
	Yes	1,359	88.2
Weekly working hours	≤ 40	577	37.5
	41–48	780	50.6
	49–58	123	8
	≥ 59	60	3.9
Number of night shifts per month	0	181	11.8
	1–4	239	15.5
	5–8	659	42.8
	> 8	461	29.9
Smoking	No	1,453	94.4
	Yes	87	5.6
Alcohol using	No	1,355	88
	Yes	185	12

N, Number.

### 3.2 Scale scores

The reliability of the scales used in this study was confirmed through internal consistency analysis. The ERI scale showed a Cronbach’s alpha of 0.82, indicating acceptable reliability. The Work-Family Behavioral Role Conflict Scale (WFBRC-S) demonstrated high reliability with a Cronbach’s alpha of 0.95, and the SSD-CN had a Cronbach’s alpha of 0.90. Among the participants, 1,240 nurses (80.5%) reported experiencing ERI (Table 1). The mean ERI score for the participants was 0.93 ± 0.57, indicating that a significant portion of the nurses experienced an imbalance between effort and reward. The average WFC score was 42.48 ± 16.2. According the results of SSD-CN, the mean overall symptom score was 39.58 ± 13.61, reflecting moderate to high levels of physical health concerns in the sample (Table 2).

**Table 2:** Evaluation scales and their scores for each dimension involved in this study.

Items	Min	Max	Score (mean ± SD)
ERI	0.2	5	0.932 ± 0.57
Effort score dimension	6	30	19.22 ± 5.35
Reward score dimension	11	55	23.72 ± 9.1
Intrinsic effort score dimension	5	25	12.6 ± 4.74
WFC	19	95	42.48 ± 16.21
Work-to-family conflict	7	35	18.27 ± 6.88
Family-to-work conflict	12	60	24.21 ± 10.75
Overall symptoms	20	80	39.58 ± 13.61
Somatic symptoms	10	40	19.13 ± 6.87
Mental symptoms	10	40	20.46 ± 7.08

ERI, Effort-Reward Imbalance; WFC, Work–Family Conflict; SD, Standard Deviation.

### 3.3 Correlation analysis

A correlation analysis was performed to examine the relationships between ERI, WFC, and health outcomes. Significant positive associations were observed between ERI and several key variables. Specifically, ERI had positive moderately strong correlation with WFC (*r* = 0.552, *p* < 0.01), overall symptoms (*r* = 0.554, *p* < 0.01), somatic symptoms (*r* = 0.547, *p* < 0.01), and mental symptoms (*r* = 0.533, *p* < 0.01). ERI was highly correlated with intrinsic effort (*r* = 0.712, *p* < 0.01). These results indicated that higher levels of ERI were consistently associated with higher levels of WFC and greater health-related symptoms (Table 3).

**Table 3:** Correlation analysis between ERI, WFC, and health outcomes.

Items	ERI	WFC	Overall symptoms	Somatic symptoms	Mental symptoms	Intrinsic effort
ERI	1					
WFC	0.552^**^	1				
Overall symptoms	0.554^**^	0.655^**^	1			
Somatic symptoms	0.547^**^	0.660^**^	0.976^**^	1		
Mental symptoms	0.533^**^	0.618^**^	0.975^**^	0.903^**^	1	
Intrinsic effort	0.712^**^	0.632^**^	0.632^**^	0.649^**^	0.583^**^	1

**p < 0.01. The correlation was significant. ERI, Effort-Reward Imbalance; WFC, Work–Family Conflict.

### 3.4 Mediation effect of WFC on the relationship between ERI and health outcomes

A mediation analysis was conducted using Hayes’ PROCESS macro (Model 4) to examine whether WFC mediated the relationship between ERI and health outcomes. ERI had a direct effect on overall symptoms (*β* = 0.554, *t* = 26.080, *p* < 0.01), somatic symptoms (*β* = 0.533, *t* = 24.702, *p* < 0.01), and mental symptoms (*β* = 0.547, *t* = 25.640, *p* < 0.01). When WFC was included as a mediator, the direct predictive effect of ERI on overall symptoms (*β* = 0.276, *t* = 12.543, *p* < 0.01), somatic symptoms (*β* = 0.276, *t* = 11.999, *p* < 0.01), and mental symptoms (*β* = 0.263, *t* = 11.956, *p* < 0.01) remained significant. ERI had a significant positive predictive effect on WFC (*β* = 0.552, *t* = 25.979, *p* < 0.01). And the WFC also had positive predictive effect on overall symptoms (*β* = 0.503, *t* = 22.834, *p* < 0.01), physical symptoms (*β* = 0.465, *t* = 20.215, *p* < 0.01), and mental symptoms (*β* = 0.515, *t* = 23.420, *p* < 0.01; Table 4).

**Table 4:** Regression analysis of the mediating role model of WFC.

	Model 1		Model 2		Model 3	
	*β*	*t*	*β*	*t*	*β*	*t*
Overall symptoms						
ERI	0.554	26.080**	0.552	25.979**	0.276	12.543**
WFC					0.503	22.834**
Somatic symptoms						
ERI	0.533	24.702**	0.552	25.979**	0.276	11.999**
WFC					0.465	20.215**
Mental symptoms						
ERI	0.547	25.640**	0.552	25.979**	0.263	11.956**
WFC					0.515	23.420**

**p < 0.01. Model 1: ERI predicting symptoms; Model 2: ERI predicting WFC; Model 3: ERI and WFC collaborative predicting symptoms. ERI, Effort-Reward Imbalance, WFC, Work–Family Conflict.

The bootstrap 95% CI for the direct effects of ERI on overall symptoms, somatic symptoms, and mental symptoms, as well as the mediating effects of WFC, did not include 0, indicating that ERI could not only directly predict overall symptoms (*β* = 0.276), somatic symptoms (*β* = 0.276), and mental symptoms (*β* = 0.263), but also predict overall symptoms (*β* = 0.278), somatic symptoms (*β* = 0.257), and mental symptoms (*β* = 0.284) through the mediating effects of WFC. The models of the Bootstrap method path effect test we used also confirm these results (Table 5).

**Table 5:** The relationship between WFC, ERI and health outcomes.

Pathways	*β*	Boot SE	95%*CI*	
			LLCI	ULCI
Overall symptoms				
Total effect	0.554	0.021	0.512	0.595
Direct effect	0.276	0.022	0.233	0.319
Indirect effect	0.278	0.019	0.242	0.314
Bootstrap				
ERI → WFC	0.552	0.035	0.49	0.63
WFC → Overall Symptoms	0.276	0.031	0.218	0.339
ERI → Overall Symptoms	0.503	0.028	0.449	0.557
Somatic symptoms				
Total effect	0.533	0.022	0.491	0.575
Direct effect	0.276	0.023	0.231	0.321
Indirect effect	0.257	0.019	0.222	0.295
Bootstrap				
ERI → WFC	0.552	0.035	0.488	0.628
WFC → Somatic Symptoms	0.465	0.028	0.411	0.519
ERI → Somatic Symptoms	0.276	0.029	0.22	0.334
Mental symptoms				
Total effect	0.547	0.021	0.505	0.589
Direct effect	0.263	0.022	0.22	0.306
Indirect effect	0.284	0.02	0.246	0.324
Bootstrap				
ERI → WFC	0.552	0.034	0.493	0.626
WFC → Mental Symptoms	0.263	0.032	0.205	0.331
ERI → Mental Symptoms	0.515	0.029	0.458	0.57

The table showed the total effect, direct effect, indirect effect, and Bootstrap path effect test. WFC, Work–Family Conflict; ERI, Effort-Reward Imbalance; SE, Standard Error; CI, Confidence Interval; LLCI, Lower Limit of the Confidence Interval; ULCI, Upper Limit of the Confidence Interval.

### 3.5 Moderation effect of intrinsic effort on the relationship between ERI and WFC

Moderation analysis was conducted using Hayes’ PROCESS macro (Model 7) to assess whether intrinsic effort moderated the effect of ERI on WFC. After incorporating intrinsic effort into the model, we found that the interaction term between ERI and intrinsic effort had a significant predictive effect on WFC (*β* = −0.104, *p* < 0.01). This result indicated that intrinsic effort could regulate the predictive effect of ERI on WFC (Table 6).

**Table 6:** Analysis of the moderating effect of intrinsic effort.

Items	*β*	SE	*t*	95%CI	
				LLCI	ULCI
WFC	0.074	0.023	3.271	0.029	0.118
ERI	0.431	0.045	9.572	0.343	0.519
Intrinsic Effort	0.392	0.031	12.631	0.331	0.453
ERI × Intrinsic Effort	−0.104	0.017	−6.238	−0.136	−0.071

**p < 0.01. WFC, Work–Family Conflict; ERI, Effort-Reward Imbalance; SE, Standard Error; CI, Confidence Interval; LLCI, Lower Limit of the Confidence Interval; ULCI, Upper Limit of the Confidence Interval.

**Figure 1:**
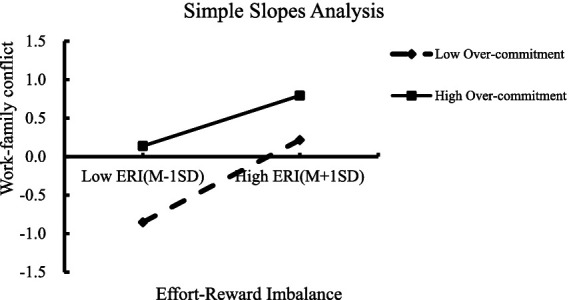
Simple slopes analysis of intrinsic effort. As the over commitment decreased from low to high, the slope decreased. This meant that as the level of intrinsic effort increased, the moderating effect of ERI on WFC gradually decreased. ERI, Effort-Reward Imbalance; WFC, Work–Family Conflict; M, Mean; SD, Standard Deviation.

Simple slopes analysis revealed that at lower levels of intrinsic effort (M - 1SD), ERI has a significant positive moderating effect on WFC (simple slope = 0.535, *t* = 9.046, *p* < 0.01). In contrast, at higher levels of intrinsic effort (M + 1SD), the effect of ERI on WFC was weaker (simple slope = 0.328, *p* < 0.001). The result showed that as the level of intrinsic effort increased, the moderating effect of ERI on WFC gradually decreased (Figure 1).

### 3.6 Combined effects on health outcomes

**Figure 2:**
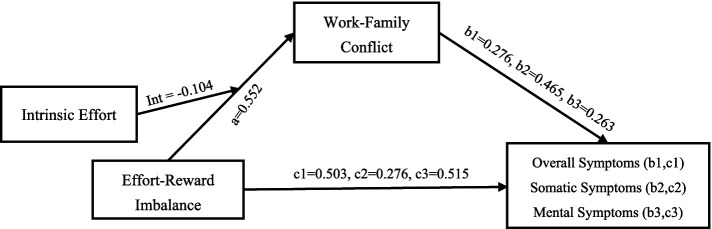
The moderating effect of intrinsic effort between ERI and WFC. Both ERI and WFC couldn’t directly affect the health outcomes of emergency nurses, and ERI could also act on WFC to affect health outcomes. Intrinsic effort could mediate the moderating effect of the relationship between ERI and WFC, thereby potentially influencing health outcomes. ERI, Effort-Reward Imbalance; WFC, Work–Family Conflict.

At the three levels of intrinsic effort (M-1SD, M, M + 1SD) in the combined models, the moderating effect of WFC between ERI and overall symptoms, somatic symptoms, and mental symptoms also showed a decreasing trend. The results indicated that as the level of intrinsic effort of emergency nurses decreased, ERI was more likely to trigger their overall symptoms, somatic symptoms, and mental symptoms through WFC among them. As ERI increases, the level of intrinsic investment also rises. In low-level intrinsic investment, as ERI increases, conflict escalation becomes more significant. As the internal investment of ED nurses decreases, ERI is more likely to exacerbate WFC, leading to physical and mental symptoms (Tables 2, 7; Figure 2).

**Table 7:** Mediating effects at different levels of intrinsic effort.

Items	*β*	SE	95%*CI*	
			LLCI	ULCI
Overall symptoms				
Eff1(*M*^-1SD^)	0.269	0.034	0.202	0.337
Eff2(*M*)	0.217	0.026	0.165	0.269
Eff3(*M*^+1SD^)	0.165	0.02	0.125	0.205
Eff2- Eff1	−0.052	0.01	−0.072	−0.034
Eff3- Eff1	−0.104	0.019	−0.145	−0.068
Eff3- Eff2	−0.052	0.01	−0.072	−0.034
Moderated mediation effect	−0.052	0.01	−0.072	−0.034
Somatic symptoms				
Eff1(*M*^-1SD^)	0.249	0.032	0.188	0.314
Eff2(*M*)	0.201	0.025	0.153	0.252
Eff3(*M*^+1SD^)	0.152	0.019	0.116	0.19
Eff2- Eff1	−0.048	0.009	−0.067	−0.031
Eff3- Eff1	−0.096	0.018	−0.134	−0.062
Eff3- Eff2	−0.048	0.009	−0.067	−0.031
Moderated mediation effect	−0.048	0.009	−0.067	−0.031
Mental symptoms				
Eff1(*M*^-1SD^)	0.249	0.032	0.188	0.314
Eff2(*M*)	0.201	0.025	0.153	0.252
Eff3(*M*^+1SD^)	0.152	0.019	0.116	0.19
Eff2- Eff1	−0.048	0.009	−0.067	−0.031
Eff3- Eff1	−0.096	0.018	−0.134	−0.062
Eff3- Eff2	−0.048	0.009	−0.067	−0.031
Moderated mediation effect	−0.048	0.009	−0.067	−0.031

M, Mean; SD, Standard Deviation; SE, Standard Error; CI, Confidence Interval; LLCI, Lower Limit of the Confidence Interval; ULCI, Upper Limit of the Confidence Interval.

## 4 Discussion

This study explored the relationships between ERI, WFC, and health outcomes among emergency nurses, with a focus on the moderating role of intrinsic effort. The findings provided valuable insights into occupational health in the high-stress environment of EDs and suggested potential interventions to improve nurse well-being.

### 4.1 ERI and health outcomes

The study confirmed that ERI was associated with both somatic and mental health symptoms, highlighting the impact of occupational stress on nurse health outcomes. The high prevalence of ERI among emergency nurses indicated the need for addressing this issue in high-demand healthcare environments (30, 31). Previous studies linked ERI to various adverse health outcomes, including cardiovascular diseases and emotional exhaustion (32–34). Our findings extended this knowledge by demonstrating that the stress resulting from ERI in the ED setting might worsen physical and mental health outcomes over time (35, 36). This was consistent with the recent cross-sectional study conducted by Shi Y et al., which included hospitals in a province of China, but our study had a larger sample size and covered hospitals across the country (37).

The strength of the direct association between ERI and health outcomes suggested that interventions aimed at improving workplace conditions, such as better compensation, recognition, and workload management, should be prioritized. The persistence of this direct effect, even after accounting for WFC, implied that addressing ERI at its root may significantly reduce the health burden on emergency nurses.

### 4.2 WFC as a mediator

The findings also showed that WFC mediated the relationship between ERI and nurse health outcomes. Our results continued the previous findings of Nigatu et al. and Wang et al. (38, 39). Nurses experiencing ERI were more likely to encounter conflicts between their professional and personal lives, which exacerbated stress and worsened health outcomes. This effect was particularly relevant in healthcare settings like EDs, where long and irregular working hours were common (40).

These results had important implications for workplace policies (26, 41). While addressing ERI was essential, strategies to reduce WFC should also be considered. Introducing flexible work schedules, telecommuting options, and family-supportive policies might help mitigate the negative effects of ERI on nurse health. Researches showed that such interventions could improve job satisfaction and reduce burnout (42, 43).

### 4.3 The role of intrinsic effort

An important finding of this study was the moderating effect of intrinsic effort. Nurses who invested moderate levels of emotional and psychological effort in their work appeared to be protected from the negative effects of ERI, as this effort provided a sense of purpose and fulfillment (44). In this case, similar to a study conducted in Switzerland, we found that hard work can alleviate some of the effects of ERI (35). However, when intrinsic effort became excessive, it might no longer served as a protective factor (45). Instead, high levels of emotional investment might exacerbate the effects of ERI, increasing the likelihood of WFC and negatively impacting health. Previously, a similar study conducted on nurses in the United States also supported our findings with similar trends in results (46). This finding suggested that there was an optimal level of intrinsic effort that helped nurses manage work-related stress without overcommitting themselves emotionally (47, 48). Future research should investigate how to balance emotional engagement in work to prevent burnout while maintaining job satisfaction.

### 4.4 Implications for healthcare management

These results offered several important implications for healthcare management. First, addressing ERI through workplace interventions aimed at improving compensation, workload distribution, and recognition was crucial. Such interventions might alleviate the health burden associated with ERI in emergency nurses (49). Second, reducing WFC through flexible scheduling, childcare support, and policies that promoted work-life balance could significantly improve nurse well-being. This was particularly relevant in a profession where many workers bore significant family responsibilities (50).

Finally, managing intrinsic effort should be a focus for healthcare managers. Encouraging emotional investment in work while providing resources to avoid overcommitment, such as resilience training and stress management programs, might help nurses maintain a healthy balance between their professional and personal lives (51). These interventions could ultimately improve both nurse retention and the quality of care delivered to patients.

### 4.5 Strengths of this study

This study has several strengths that contribute to its significance in the field of occupational health. First, this study addressed an understudied yet critical population, namely emergency nurses. Due to the demanding nature of their work, they were at high risk of occupational stress. By focusing on this group, the study provided valuable insights that were directly applicable to healthcare management and policy-making. Second, the use of validated instruments, such as the ERI scale and WFBRC-S, ensured the reliability of the measurements and supported the generalizability of the findings to similar healthcare settings. Third, the large sample size and the inclusion of nurses from multiple tertiary hospitals across different regions in China enhanced the study’s external validity, making the findings more robust. Compared to earlier international studies with sample sizes of only a few hundred cases, our sample size was relatively more convincing (52). Finally, this study is one of the few to examine the complex interaction between ERI, WFC, and intrinsic effort. Although previous studies have been reported on the ERI of Chinese nurses, they have not delved into the relationship between the three, especially without considering the important role of intrinsic effort (12). Our study offered a novel perspective on how emotional investment in work could have both protective and detrimental effects on health.

### 4.6 Limitations and future directions

This study has several limitations. First, the cross-sectional design precluded the establishment of causal relationships between ERI, WFC, and health outcomes (53). Future longitudinal studies are needed to explore these relationships over time. Second, the study was conducted in EDs in mainland China, and the findings might not be generalizable to other healthcare settings or cultural contexts. Cross-cultural research is necessary to validate these findings and assess how cultural factors influence the ERI-WFC relationship.

In addition, the reliance on self-reported data introduced potential bias. Although we fully respected the originality of the data, there might still be potential biases in statistical analysis. Finally, due to the scarcity of similar studies, there were relatively few studies available for reference and comparison internationally, and we could only focus on the level of public hospitals in China, making it difficult to horizontally compare with the situation of emergency nurses in global hospitals. Future studies should incorporate objective measures, such as physiological stress markers or third-party assessments, to strengthen the reliability of the results.

## 5 Conclusion

This study demonstrated a significant association between ERI and adverse health outcomes in emergency nurses, with WFC mediating this relationship. Intrinsic effort moderated the impact of ERI, with moderate emotional investment buffering its negative effects, while excessive effort increased the risk of burnout. These findings highlighted the need for targeted interventions that address both ERI and WFC through improved workplace conditions and supportive policies. Future research should focus on longitudinal effects and intervention strategies to enhance the well-being of nurses in high-pressure healthcare environments.

## Data Availability

The raw data supporting the conclusions of this article will be made available by the authors, without undue reservation.
